# The Wheat MYB Transcription Factor TaMYB^31^ Is Involved in Drought Stress Responses in *Arabidopsis*

**DOI:** 10.3389/fpls.2018.01426

**Published:** 2018-09-28

**Authors:** Yue Zhao, Xiyong Cheng, Xiaodan Liu, Huifang Wu, Huihui Bi, Haixia Xu

**Affiliations:** ^1^College of Life Sciences, Henan Agricultural University, Zhengzhou, China; ^2^College of Agronomy/Collaborative Innovation Center of Henan Grain Crops/National Key Laboratory of Wheat and Maize Crop Science, Henan Agricultural University, Zhengzhou, China

**Keywords:** wheat, MYB, drought, transgenic, *Arabidopsis*, RNA-seq

## Abstract

Drought is one of the major environmental stresses limiting crop growth and production. MYB family transcription factors play crucial roles in response to abiotic stresses. Previous studies found that *TaMYB31* is transcriptionally induced by drought stress. However, the biological functions of *TaMYB31* in drought stress responses remained unknown. In this study, three *TaMYB31* homoeologous genes from hexaploid wheat, designated *TaMYB31-A, TaMYB31-B*, and *TaMYB31-D*, were cloned and characterized. Expression analysis showed that *TaMYB31* genes have different tissue expression patterns, and *TaMYB31-B* has relatively high expression levels in most tested tissues. All the three homoeologs were up-regulated by polyethylene glycol (PEG) 6000 and abscisic acid (ABA) treatments. Subcellular localization analyses revealed that TaMYB31 is localized to the nucleus. Ectopic expression of the *TaMYB31-B* gene in *Arabidopsis* affected plants growth and enhanced drought tolerance. In addition, seed germination and seedling root growth of *TaMYB31-B* transgenic plants were more sensitive to exogenous ABA treatment compared to wild type control. RNA-seq analysis indicated that TaMYB31 functions through up-regulation of wax biosynthesis genes and drought-responsive genes. These results provide evidence that TaMYB31 acts as a positive regulator of drought resistance, and justify its potential application in genetic modification of crop drought tolerance.

## Introduction

Drought is a major environmental stress that limits crop growth and production. A large proportion of agriculturally productive areas worldwide have been seriously affected by enduring droughts, which have led to serious damage to crops (Kudo et al., 2017). It was calculated that drought has caused an average of 13.7% reduction in cereal production worldwide over the past few decades (Lesk et al., 2016). Moreover, the world population continues to grow rapidly, therefore, to sustain such a large population, it is essential to enhance crop drought tolerance. Unraveling the molecular and biochemical mechanisms which enable plants to cope with environmental challenges is of great importance to improve stress tolerance and yield (Hrmova and Lopato, 2014).

The molecular mechanisms underlying plant drought tolerance have been investigated intensively. In fact, plants respond to drought via various dynamic regulatory networks, in which transcription factors (TFs) play important roles (Krasensky and Jonak, 2012). A single TF could activate and/or repress the expression of a number of target genes through direct binding to its specific DNA recognition sequence, also known as *cis*-acting element, present in the promoters of its downstream genes. So far, several TF families, such as MYB, WRKY, AP2/ERF, NAC, and HD-ZIP, have been reported to act as master regulators in response to drought stress in plants (Sakuma et al., 2006; Ding et al., 2009; Nuruzzaman et al., 2013; Banerjee and Roychoudhury, 2017).

MYB TFs constitute one of the largest TF families and have conserved MYB DNA-binding domain containing 52 amino acids. The MYB TF family consists of four major subfamilies differing in the number of imperfect repeat of R1-R3 DNA-binding domains: 1R-MYB or MYB-related proteins, R2R3-MYB, 3R-MYB (R1R2R3-MYB), and 4R-MYB. In particular, R2R3-MYB TFs regulate various plant developmental processes, such as epidermal cell, cuticle, trichome and stomata formation, as well as a series of environmental stresses including drought (Dubos et al., 2010). For example, increased expression of *Arabidopsis MYB15* resulted in enhanced drought and salt tolerance and hypersensitivity of transgenic *Arabidopsis* to exogenous ABA (Ding et al., 2009). Overexpression of the drought-induced *AtMYB41* improved drought tolerance in transgenic *Arabidopsis* (Cominelli et al., 2008; Lippold et al., 2009). *AtMYB96* was demonstrated to play important roles in increasing cuticular wax deposition and improving drought tolerance in *Arabidopsis* (Seo et al., 2009, 2011). Overexpression of the rice *OsMYB48-1* conferred drought tolerance to transgenic rice plants (Xiong et al., 2014). Heterologous expression of *OsMYB55* led to improved tolerance to drought and heat stresses in transgenic maize (Casaretto et al., 2016).

In our previous work, six cuticle biosynthesis-related wheat MYB genes were cloned. As an ortholog of *AtMYB96*, the drought inducible *TaMYB31* has been suggested to be involved in the regulation of drought responses (Bi et al., 2016). However, the biological function of *TaMYB31* has not yet been investigated. In this study, we cloned *TaMYB31* homoeologous genes (*TaMYB31-A, TaMYB31-B*, and *TaMYB31-D*) from hexaploid wheat, analyzed their expression patterns, and investigated their functions via heterologous overexpression and RNA-seq analysis. The three homoeologous genes displayed differential expression in various wheat organs, and ectopic overexpression of *TaMYB31-B* in *Arabidopsis* increased drought tolerance. The improved drought tolerance was at least partly due to up-regulation of wax biosynthesis genes and drought responsive genes. Our results shed light on the potential value of *TaMYB31* in genetic improvement of crop drought tolerance.

## Materials and methods

### Plant materials and growth conditions

The hexaploid wheat Chinese Spring and three diploid ancestor wheats, the *Triticum urartu* accession UR206, the *Aegilops tauschii* accession Y2282 and the *Aegilops speltoides* accession Y2006, were used to isolate the genomic and ORF sequences of *TaMYB31* genes. For gene expression analyses under abiotic stresses, 10-day-old wheat seedlings were treated with 20% polyethylene glycol (PEG) 6000 and 200 μM ABA, and harvested at 0, 0.5, 1, 2, 4, 6, 12, and 24 h. Each sampled material was immediately rinsed in sterile distilled water, dried with filter papers, then frozen in liquid nitrogen and stored at −80°C for further analyses. To analyze tissue-specific expression patterns, roots, stems, leaves, young spikes, and flowering spikes were harvested from Chinese Spring wheat and stored at −80°C.

The *Arabidopsis* genotype Col-0 was used to generate *TaMYB31-B* transgenic plants. The plants were grown in soil in a greenhouse with a constant 22°C, 50–70% relative humidity and a 16/8 h light/dark cycle at 100 μmol m^−2^s^−1^ or on 1/2MS medium. Images of rosette leaves were taken after 4 weeks grown in soil and rosette diameter was measured using ImageJ software (http://imagej.nih.gov/ij/). Plant height and fresh weight were recorded at mature period.

### Isolation of ORF and genomic sequences

Genomic sequences of *TaMYB31* homoeologous genes were retrieved from IWGSC database^1^ using *TaMYB31* ORF sequence (GenBank: KU674897.1) as a query. The acquired sequences were then used to design primers. Genomic DNA was extracted from young leaves using the CTAB method. The PCR conditions for amplifying *TaMYB31s* were as follows: 5 min at 98°C; 36 cycles of 20 s at 98°C, 20 s at 60°C, and 90 s at 72°C; and then 5 min at 72°C. The obtained purified PCR products were cloned into the pEASY-Blunt (TransGen, Beijing, China) cloning vector for sequencing. Total RNA was isolated from leaves using the TRIzol reagent following the manufacturer's protocol (TransGen, Beijing, China) and followed by treatment with DNase I (TaKaRa, Dalian, China). cDNA synthesis was conducted with 2 micrograms of purified total RNA using M-MLV reverse transcriptase (Promega, Madison, USA) according to the manufacturer's instructions. Based on the acquired genomic sequences, primers were designed to amplify the *TaMYB31* ORF sequences from wheat cv. Chinese Spring. All of the primers used are listed in Table S1.

Primers were designed using DNAMAN software^2^ Multiple sequence alignment of MYB proteins obtained in this study was performed with ClustalW (Larkin et al., 2007). Phylogenetic tree was constructed using MEGA 6.0 software (Tamura et al., 2013) with the neighbor-joining (NJ) method.

### Quantitative RT-PCR

The quantitative RT-PCR analyses were performed using the SYBR Mix ExTaq II kit (TaKaRa, Dalian, China) and the Bio-Rad CFX96 real-time system^3^ The PCR parameters were as follows: 95°C for 4 min, followed by 40 cycles of 94°C for 20 s, 60°C for 20 s and 72°C for 20 s. For expression analysis of *TaMYB31* genes in wheat, the wheat β*-actin* gene was selected as an internal control (Hu et al., 2013). *AtActin2* gene was used as an internal control for gene expression in *Arabidopsis*. Three biological replicates and three technical replicates were used in all gene expression experiments. The relative gene expression were calculated based on the comparative CT method (Livak and Schmittgen, 2001). The primers for quantitative RT-PCR are listed in Table S1.

### Subcellular localization analysis

The GFP ORF sequence was introduced in frame to the 3′ end of the *TaMYB31-B* gene sequence, and the resulting gene fusion was subcloned into the pCAMBIA1300 vector for transient expression in leaf epidermal cells of *Nicotiana benthamiana*. After agro-infiltration, plants were allowed to grow for 2 days at 22°C with a photoperiod of 16/8 h light/dark before visualization under a confocal laser-scanning microscope (FluoView™ FV1000; Olympus).

### *Arabidopsis* transformation and stress treatments

*TaMYB31-B* ORF was amplified using primers TaMYB31-OE-F and TaMYB31-OE-R (containing attB sites) and cloned into the Gateway plant expression vector pB2GW7 to generate *p35S:: TaMYB31-B*. The transformation of the generated construct into *Arabidopsis* was performed via *A. tumefaciens* (strain GV3101) mediated floral-dip method (Clough and Bent, 1998). The transgenic seedlings were confirmed by PCR and quantitative RT-PCR.

For the drought stress treatment, water was withheld for 2 weeks from 3-week-old plants with similar size grown under normal conditions in soil in a growth room, afterwards, rewatering was applied and survival rates were measured 3 days after rewatering. For water loss rate assays, leaves were detached from 4-week-old plants grown under normal conditions, and placed on filter paper at room temperature for 1, 2, 3, 4, 5, and 6 h, and then fresh weights of the leaves at each time point were measured as described previously (Zhao et al., 2017b). Three independent experiments were performed.

For the PEG stress treatment, the seeds of wild type and three homozygous T_3_ *TaMYB31-B* transgenic lines were grown on 1/2MS media in a growth chamber. After 7 days, seedlings were transferred onto 1/2MS media with or without 10% PEG6000. After another 7 days, the plates with seedlings were photographed and the fresh weights and root lengths were measured. Seedlings were grown vertically during the entire experiment. Three independent experiments were performed.

For the seed germination assay in response to ABA, WT and transgenic seeds were sterilized and germinated on 1/2MS agar plates without or with 0.3, 0.5, or 1.0 μM ABA. After 4 days of growth, the plates with seeds/seedlings were photographed and seed germination rates were calculated. Seed germination was defined as emergence of the radicle tip through the endosperm. At least 60 seeds were used for each replicate of three independent replicates. Root elongation rate assay was performed on 1/2MS media supplemented with 0 or 10 μM ABA as described in Ling et al. (2017).

### RNA-Seq analysis

Leaves were collected from 4-week-old transgenic L2 and WT plants under drought conditions (water was withheld for 1 week from 3-week-old plants). Total RNA was extracted using Trizol reagent (TransGen, Beijing, China) and RNA integrity was checked using a Bioanalyzer 2100 (Agilent, California, USA). cDNA synthesis, libraries construction, sequencing, and raw reads analysis were performed as previously described (Zhao et al., 2017a). Approximately 2 GB clean data were produced for each sample. The clean reads generated were aligned against the reference *Arabidopsis* genome using TopHat2 (Kim et al., 2013). Gene expression was calculated using the Reads Per Kilobase per Million mapped reads (RPKM) method (Mortazavi et al., 2008). Gene Ontology enrichment was analyzed using the agriGO analysis tool (Tian et al., 2017). RNA-Seq data were submitted to NCBI and can be accessed under the SRA accession number SRP157487.

## Results

### Molecular characterization of the *TaMYB31* homoeologous genes

**Figure 1:**
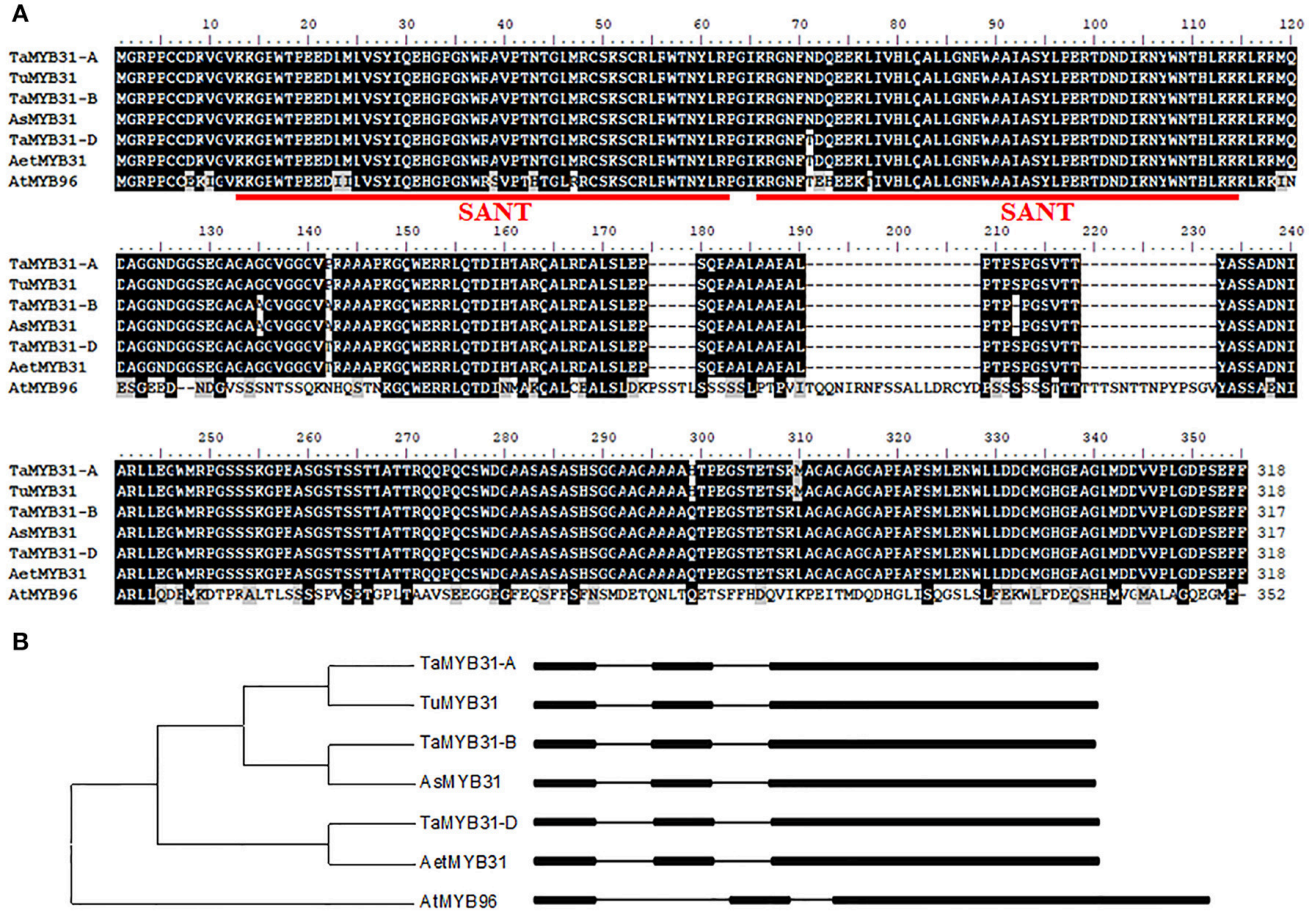
Sequence analysis of *TaMYB31* homologs. **(A)** Comparison of amino acid sequences of seven MYB31 proteins, i.e., hexaploid wheat *TaMYB31-A/B/D* (GenBank: MH428951-53), *Triticum urartu* TuMYB31 (GenBank: MH428954), *Aegilops speltoides* AsMYB31 (GenBank: MH428955), *Aegilops tauschii* AetMYB31 (GenBank: MH428956), and AtMYB96 (AT5G62470). The SANT domain is underlined. **(B)** Phylogenetic and gene structure analysis of *TaMYB31* homoeologs, three diploid ancestral wheat *MYB31* genes and *AtMYB96*. Exons and introns are represented by black boxes and lines, respectively.

To comprehensively understand the *TaMYB31* gene, the genomic and ORF sequences of *MYB31* were isolated from the diploid wheat UR206 (AA), Y2006 (SS), and Y2282 (DD) as well as hexaploid wheat Chinese Spring (BBAADD). Three homoeologs of *TaMYB31* were amplified from the hexaploid wheat, and sequence alignment indicated that the six MYB31s (three from diploid wheat and three from hexaploid wheat) obtained shared 98.4–100% identity at the amino acid level (Figure 1A). The GenBank accession numbers of the six *MYB31s* are MH428951-MH428956.

Based on the sequence alignment, the three *TaMYB* sequences were assigned to the A, B, and D genomes and accordingly named as *TaMYB31-A, TaMYB31-B*, and *TaMYB31-D*, respectively (Figure 1B). Gene structure analyses using the genomic sequences and the cDNA sequences of *TaMYB31s* revealed that the three *TaMYB31* homoeologs, *TaMYB31-A, TaMYB31-B*, and *TaMYB31-D*, contain three exons and two introns (Figure 1B).

### Expression analysis of *TaMYB31* genes in wheat

**Figure 2:**
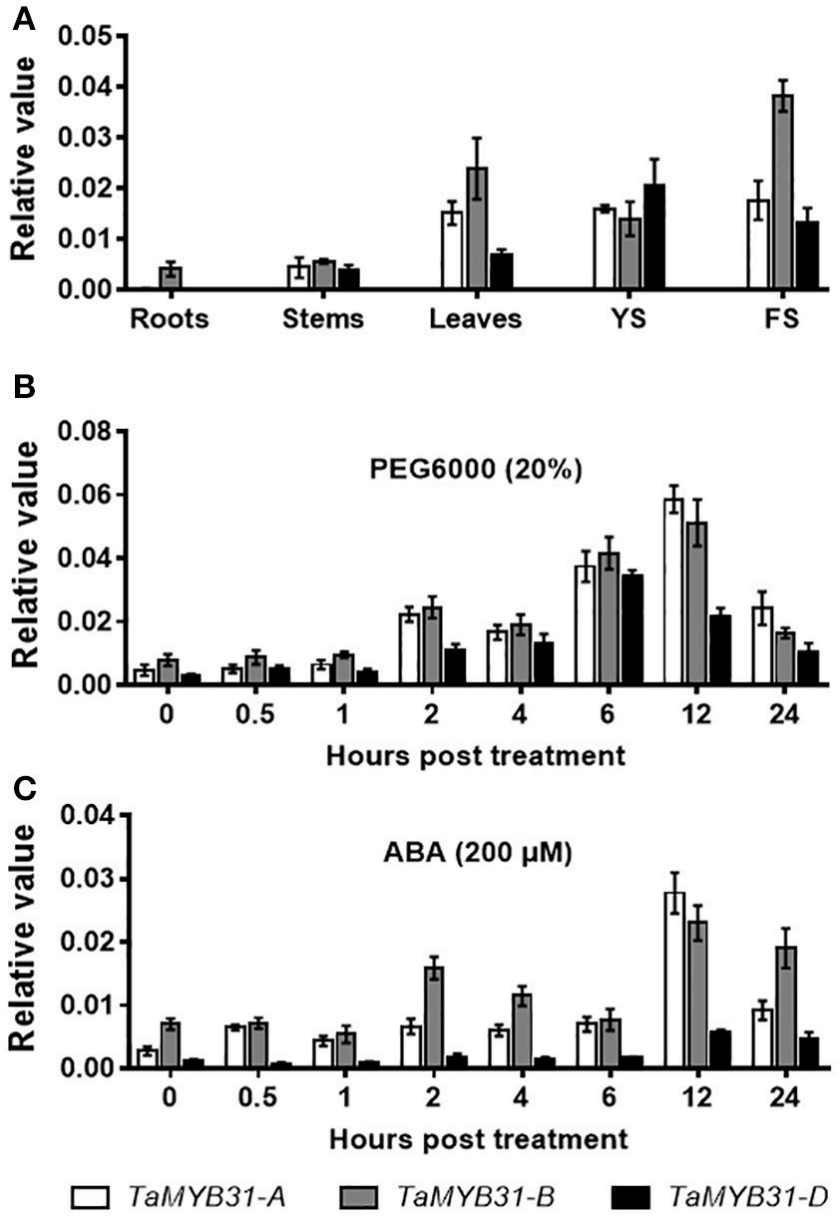
Expression patterns of *TaMYB31s*. **(A)** *TaMYB31s* expression in various wheat tissues by quantitative RT-PCR. YS, young spikes and FS, flowering spikes. Data are presented as means of three replicates ± SD. **(B,C)** Expression of *TaMYB31s* under PEG **(B)** and ABA treatment **(C)** for the indicated time points by quantitative RT-PCR. Data are presented as means of three replicates ± SD.

*TaMYB31* was demonstrated to be induced by drought stress (Bi et al., 2016), implying that this gene might play a role in the regulation of plant responses to drought stress. To unravel the biological function of the *TaMYB31* homoeologous genes, we first examined the tissue-specific expression of *TaMYB31* genes in various tissues of hexaploid wheat Chinese Spring, including roots, stems, leaves, young spikes and flowering spikes. As shown in Figure 2A, *TaMYB31* genes were strongly expressed in leaves, young spikes, and flowering spikes. Notably, *TaMYB31-A, TaMYB31-B*, and *TaMYB31-D* displayed different expression patterns, suggesting that they may play diverse roles at various developmental stages. *TaMYB31-B* had the highest transcript level in most of the tissues examined (Figure 2A), indicating that it might play a more significant regulatory role on plant development than *TaMYB31-A* and *TaMYB31-D*.

Next, the expression patterns of *TaMYB31* genes were analyzed by quantitative RT-PCR under stress conditions. Under a PEG treatment, the expression of the *TaMYB31* genes increased gradually and *TaMYB31-A* and *TaMYB31-B* peaked at 12 h, while *TaMYB31-D* peaked at 6 h (Figure 2B). As for ABA treatment, the expression of *TaMYB31-A* and *TaMYB31-D* were gradually up-regulated and peaked at 12 h (Figure 2C). The transcript of *TaMYB31-B* under ABA treatment reached a high level at 2 h and then declined rapidly and reached the untreated level at 6 h; however, *TaMYB31-B* expression increased again and reached the highest level at 12 h. In addition, the expression levels of the genes after ABA treatment appeared to be lower than that after PEG treatment. Based on the expression analyses of *TaMYB31s* in wheat tissues and under stress treatments, the *TaMYB31-B* gene was selected for further functional analysis.

### Subcellular location of the *TaMYB31-B* protein

**Figure 3:**
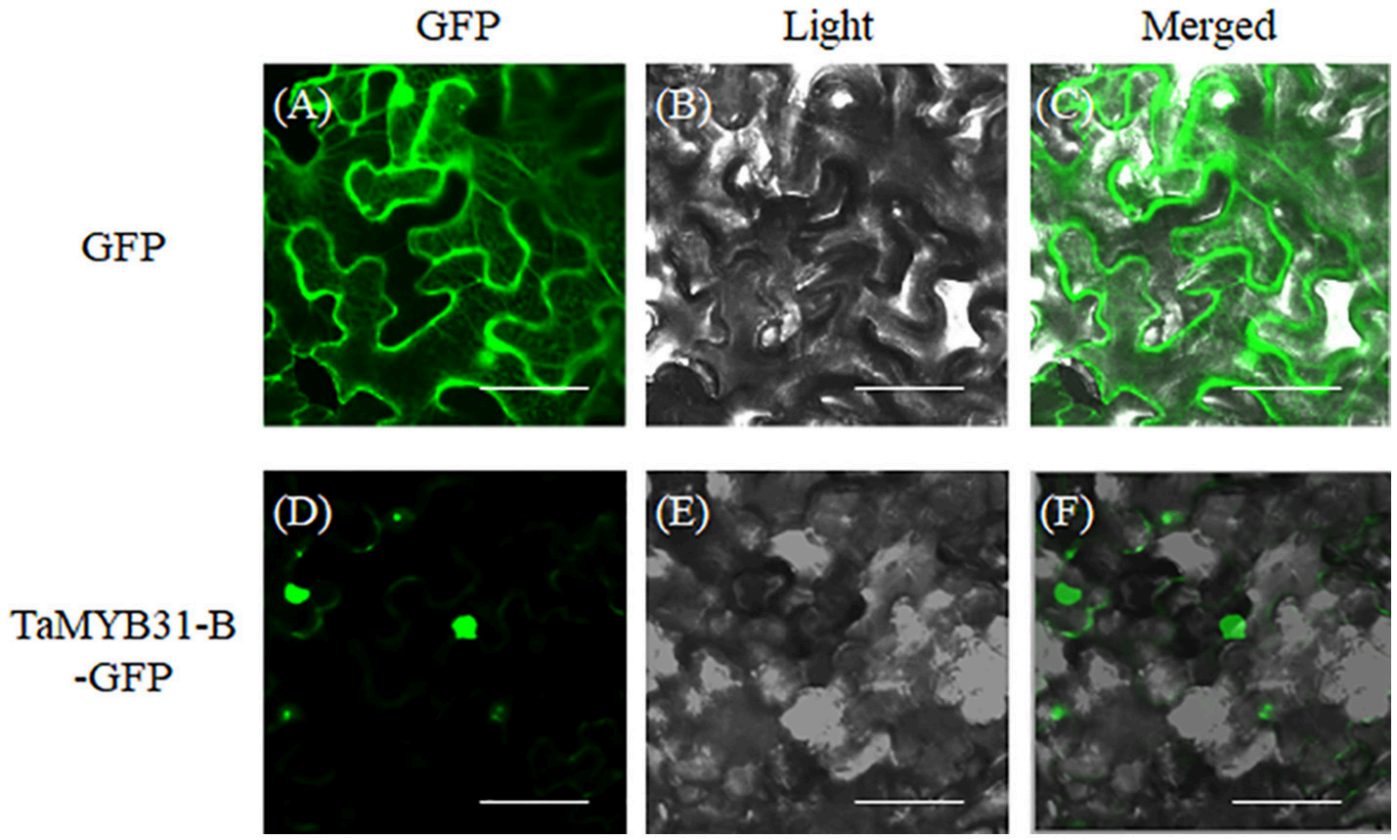
Subcellular localization of *TaMYB31-B* in *Nicotiana benthamiana* leaf epidermal cells. *35S::GFP* and *35S::TaMYB31-B-GFP* plasmids were transformed into *Nicotiana benthamiana* leaf epidermal cells and GFP fluorescence were visualized under a confocal laser-scanning microscope. From left to right, GFP fluorescence **(A,D)**, bright-field **(B,E)**, and overlays of the GFP fluorescence and bright-field **(C,F)**. Bar = 40 μm.

Nuclear localization is necessary for transcription factors to execute their functions. Subcellular localization of TaMYB31-B was investigated through transient transformation in *Nicotiana benthamiana* leaf epidermal cells. We constructed the fusion protein of TaMYB31-B with green florescent protein (GFP), and the resulting constructs containing TaMYB31-B-GFP and GFP, respectively, were introduced to *Nicotiana benthamiana* leaf epidermal cells. As shown in Figure 3, TaMYB31-B protein was localized primarily in the nucleus, while GFP was observed in both nucleus and cytosol (Figure 3).

### Overexpression of *TaMYB31-B* in *Arabidopsis*

**Figure 4:**
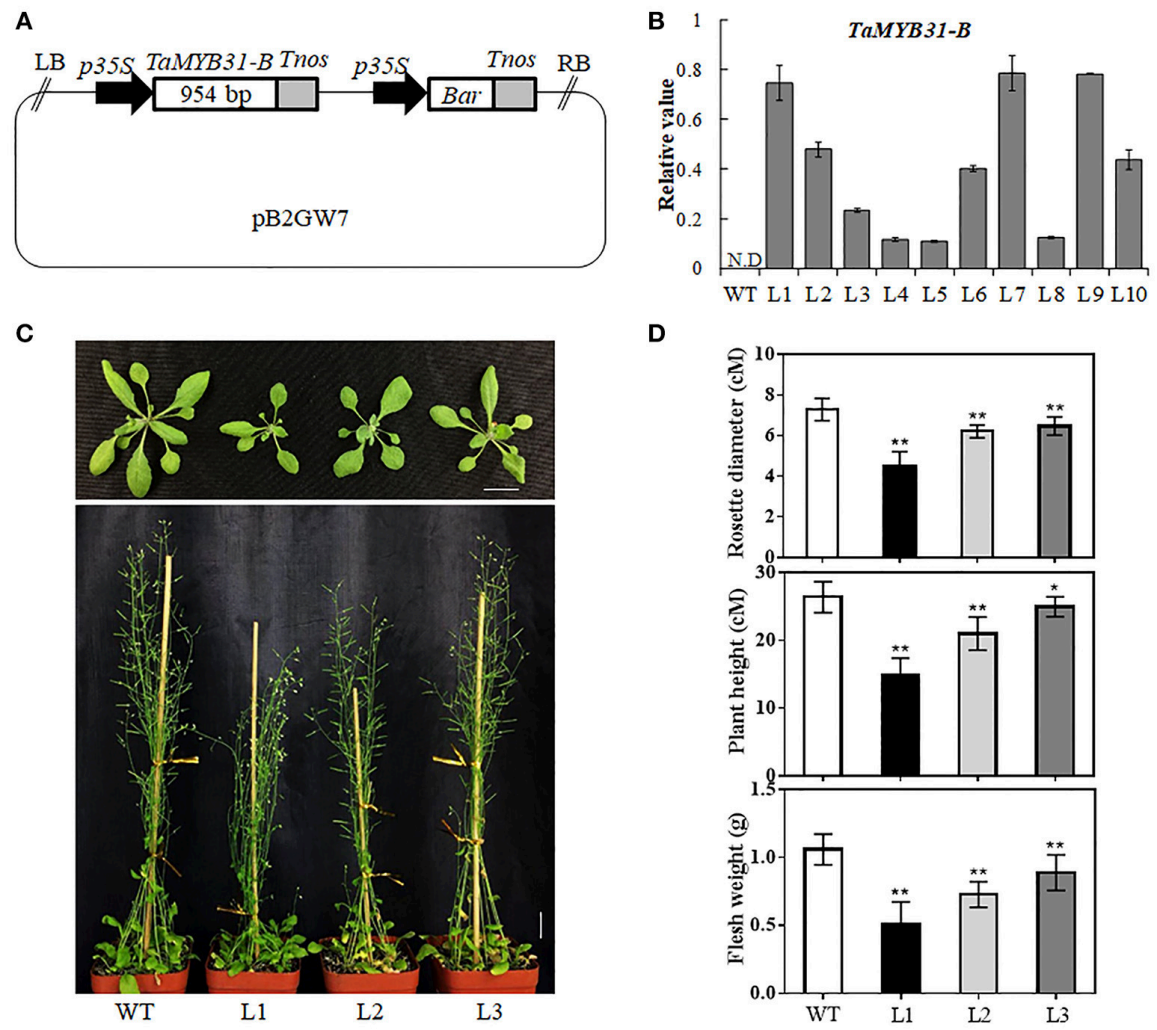
Generation of transgenic *Arabidopsis* plants overexpressing *TaMYB31-B*. **(A)** Schematic diagram of the *35S::TaMYB31-B* construct. LB left border, *p35S* cauliflower mosaic virus 35S promoter, *Tnos* nopaline synthase gene (NOS) terminator, *Bar* bialaphos-resistance gene, RB right border. **(B)** Transcript levels of *TaMYB31-B* in the leaves of T_3_ transgenic *Arabidopsis* lines via quantitative RT-PCR analysis. Bars indicate standard deviations of three replicates. WT non-transgenic plants, N.D, Not Detected. **(C)** Phenotypes of *TaMYB31-B* transgenic plants. Bar = 2 cM. **(D)** Statistical analyses of rosette diameter, plant height and fresh weight of mature plants grown in soil. ^*^ and ^**^ indicate significant differences at *P* < 0.05 and *P* < 0.01 levels, respectively, by Student's t-test.

To further dissect the biological function of *TaMYB31-B*, transgenic *Arabidopsis* plants overexpressing *TaMYB31-B* driven by *35S* promoter were generated (Figure 4A); A total of 10 independent transgenic plants were obtained, and various levels of *TaMYB31-B* gene expression were detected in T3 homozygous lines (Figure 4B). Three transgenic lines designated L1, L2, and L3 with different expression levels of *TaMYB31-B* were selected for further phenotypic assays. Under normal growth conditions, the *TaMYB31-B* transgenic plants were smaller than the wild-type plants (Figure 4C). The rosette diameter after 4 weeks of growth in soil was smaller for L1, L2, and L3 compared with the wild type (Figure 4D). Plant height and fresh weight of mature plants were reduced in the transgenic lines (Figure 4D). Notably, line L1, which had the highest expression level of *TaMYB31-B* among the three examined lines, had the smallest plant size, while L3 with the lowest transcript level of *TaMYB31-B* had the largest plant size. These results indicated that altered *TaMYB31-B* expression affects plant growth and development under normal growth conditions, possibly in a dose-dependent manner.

### Overexpression of *TaMYB31-B* increases drought tolerance

**Figure 5:**
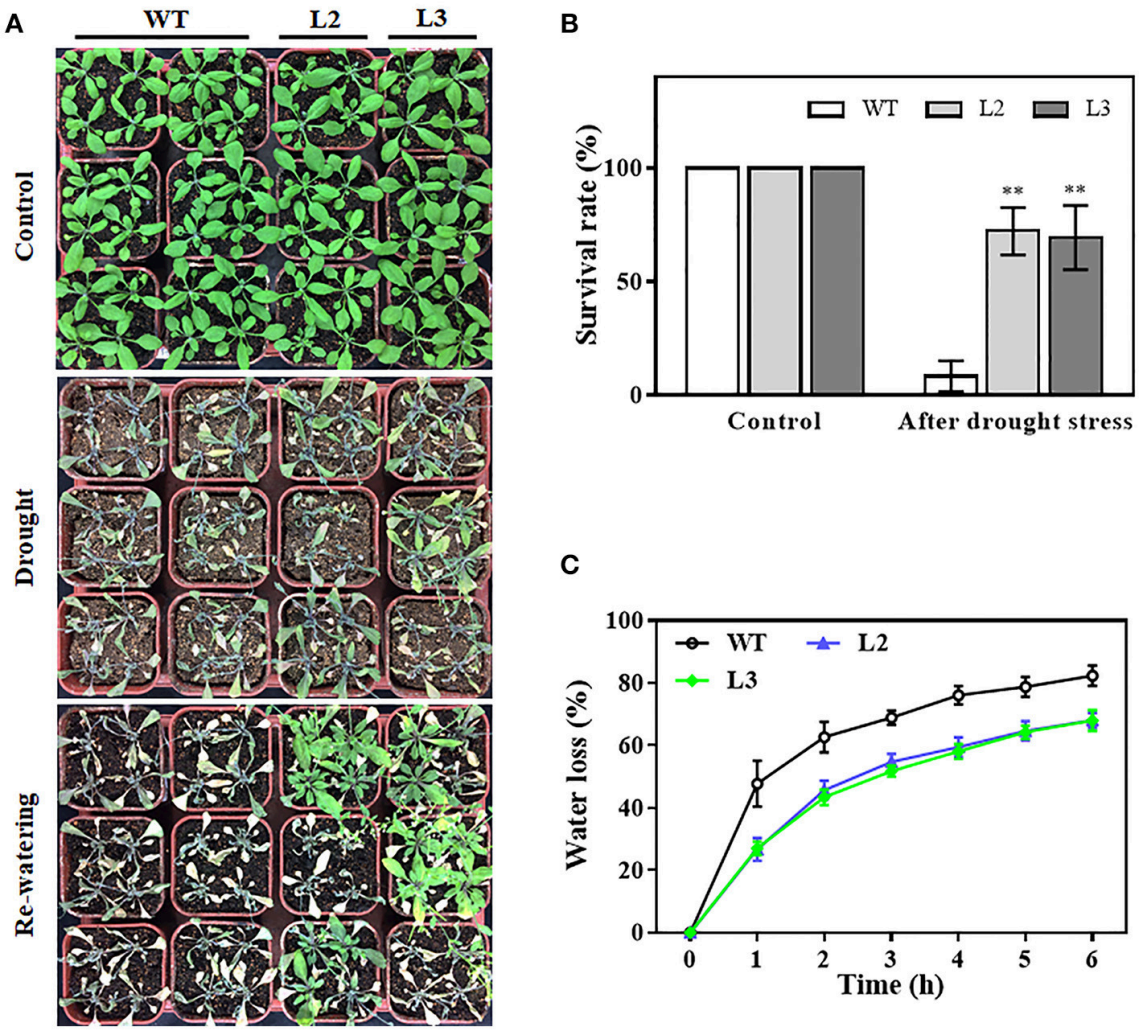
Drought tolerance responses of *TaMYB31-B* overexpressing transgenic lines. **(A)** Images of drought tolerance assays and **(B)** survival rates of WT and *TaMYB31-B* overexpressing lines of the T_3_ generation. Watering was withheld for two weeks and re-applied for 3 days. **(C)** Water loss assays in detached leaves of WT and transgenic lines. ^**^Indicates significant differences at *P* < 0.01 level by Student's *t*-test.

To evaluate the impact of *TaMYB31-B* overexpression on the drought tolerance of plants, watering was terminated for 3-week-old transgenic (L2 and L3) and wild type (WT) plants with similar size grown in soil. After 2 weeks of water deprivation, the two *TaMYB31-B* transgenic lines exhibited better growth status than WT plants (Figure 5A). The plants were then rewatered and allowed to grow for 3 days when plant survival rates were measured. The transgenic plants had significantly higher survival rates than WT plants (Figure 5B).

**Figure 6:**
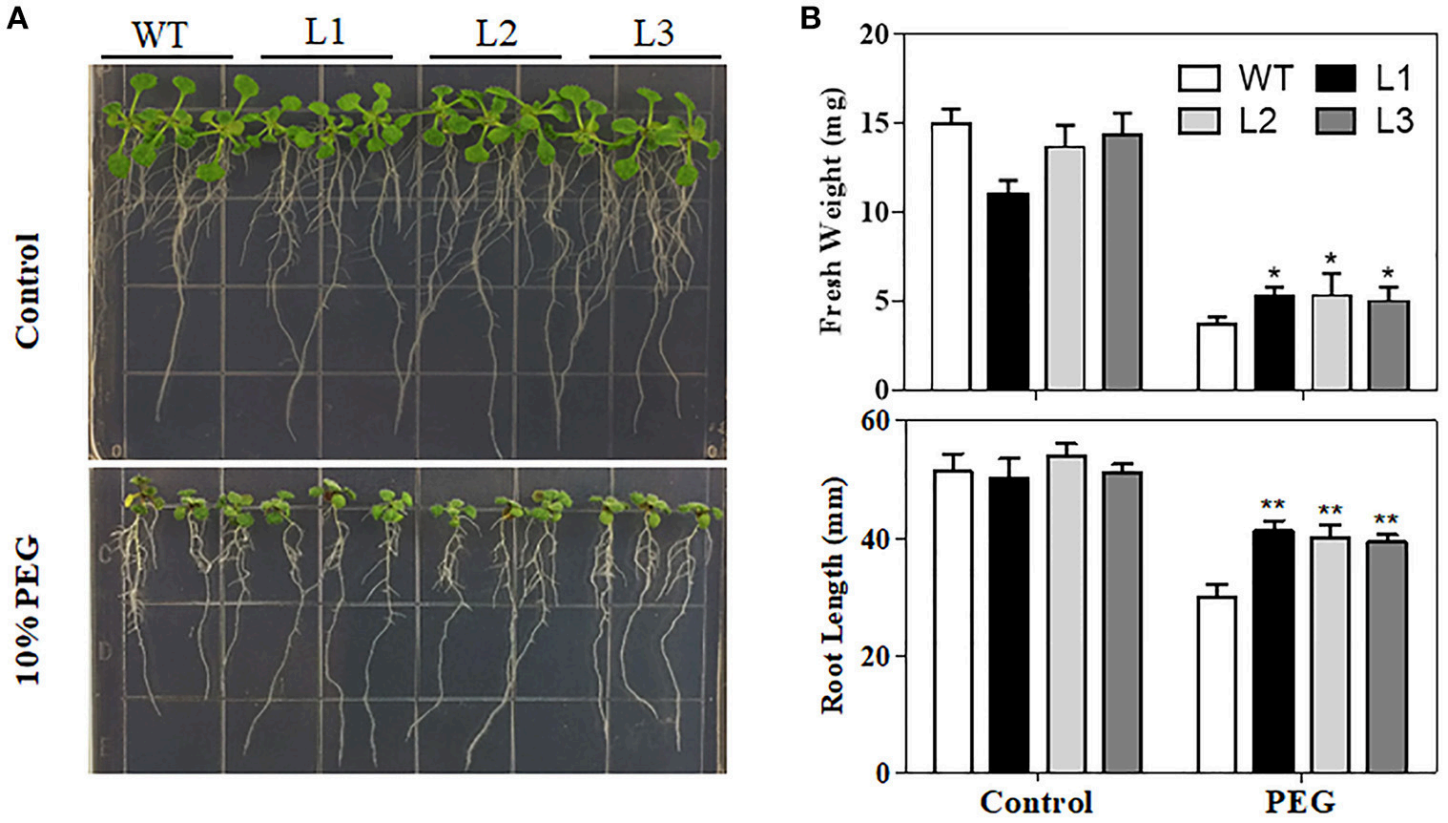
Phenotypes of WT and *TaMYB31-B* transgenic plants under PEG stress. **(A)** Images of WT and transgenic lines under PEG treatment. **(B)** Fresh weight and root lengths of *Arabidopsis* seedlings. Data are presented as means ± SD of three replicates (^*^P < 0.05, ^**^P < 0.01).

Water loss rates of detached leaves have been widely used to reflect drought tolerance in plants. Water loss rates were assessed every hour at room temperature for both transgenic plants and control plants. As shown in Figure 5C, the two transgenic lines exhibited lower rates of water loss than the control plants. Furthermore, PEG treatment, as a routine dehydration treatment, was applied; transgenic plants displayed a more tolerant phenotype in seedling growth compared to WT plants under PEG treatment (Figure 6A). The three *TaMYB31-B* transgenic lines showed higher fresh weight and longer root length than the WT under PEG conditions (Figure 6B). Taken together, these results demonstrated that *TaMYB31-B* positively regulates plant response to drought stress.

### Transgenic *Arabidopsis* plants are hypersensitive to exogenous ABA

**Figure 7:**
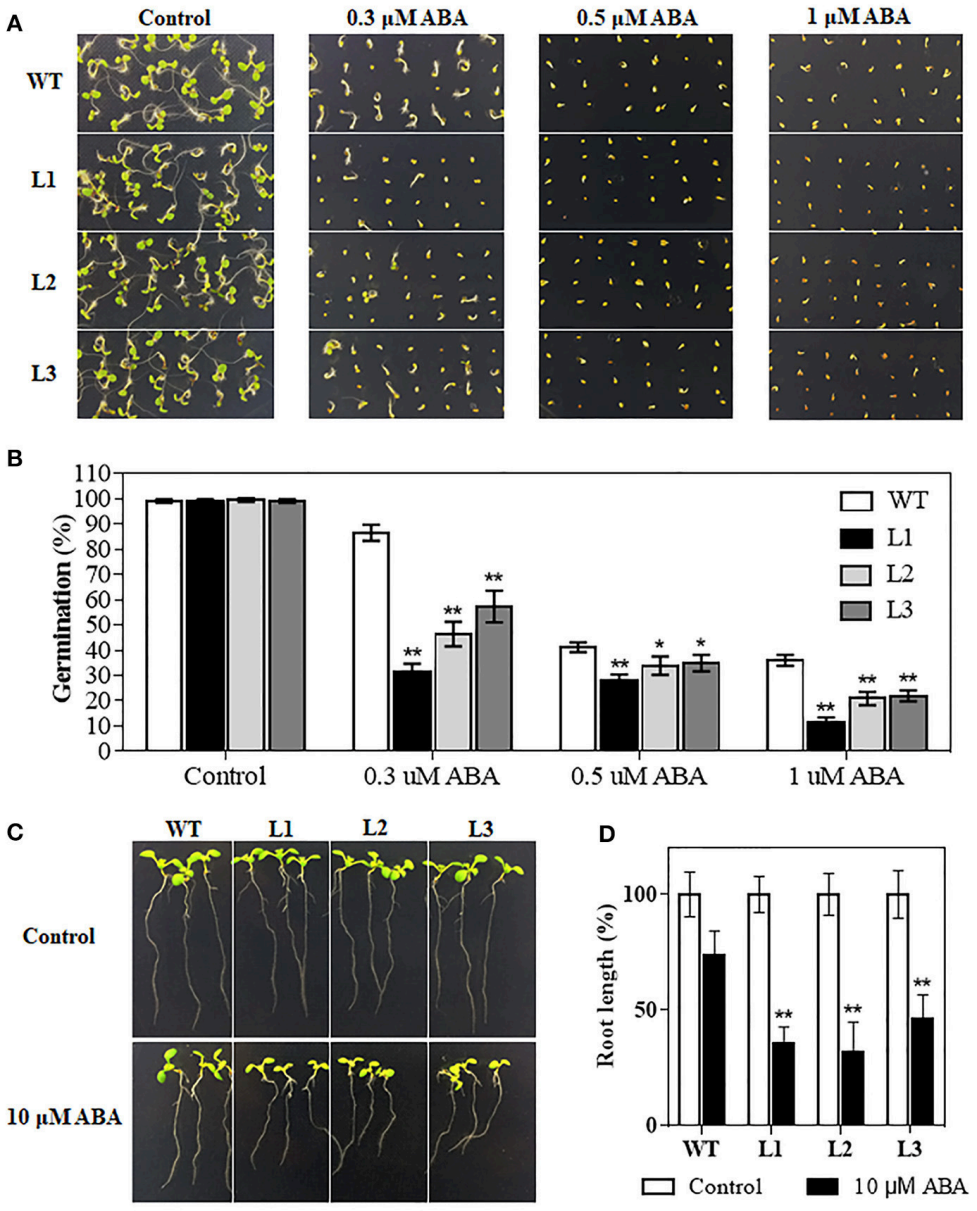
Seed germination rates analysis of WT and *TaMYB31-B* transgenic lines under ABA treatment. **(A)** Seeds of WT and three T3 *TaMYB31-B* transgenic lines grown on 1/2MS agar plates with the indicated concentrations of ABA. The photographs were taken 4 d after sowing. **(B)** Seed germination rates recorded before photographing. **(C)** Morphology of WT and transgenic seedlings on plates without or with 10 μM ABA. Images were taken 3 d after seedlings were transferred to the shown plates. **(D)** Root elongation rates of WT and transgenic seedlings at 3 d after being transferred to the plates without or with 10 μM ABA. Error bars indicate SD of three independent experiments (^*^P < 0.05, ^**^P < 0.01).

The plant hormone ABA mediates plant development and various abiotic stresses. *TaMYB31-B* expression was significantly induced by ABA (Figure 2C). We therefore investigated whether *TaMYB31* plays a role in ABA signaling pathway. Homozygous transgenic lines (L1, L2, and L3) and WT plants were selected and treated with exogenous ABA. As shown in Figures 7A,B, germination of the transgenic lines and WT was similar on ABA-free medium (Control), whereas transgenic lines had inferior germination on media containing 0.3, 0.5, or 1μM ABA compared to WT plants. Furthermore, the transgenic seedlings grown on 10 μM ABA showed reduced root elongation compared with that of the WT seedlings (Figures 7C,D). These observations indicated that *TaMYB31-B* overexpressing plants are hypersensitive to ABA during seed germination and seedling root growth.

### Transcriptome analysis of *TaMYB31-B* transgenic *Arabidopsis*

**Figure 8:**
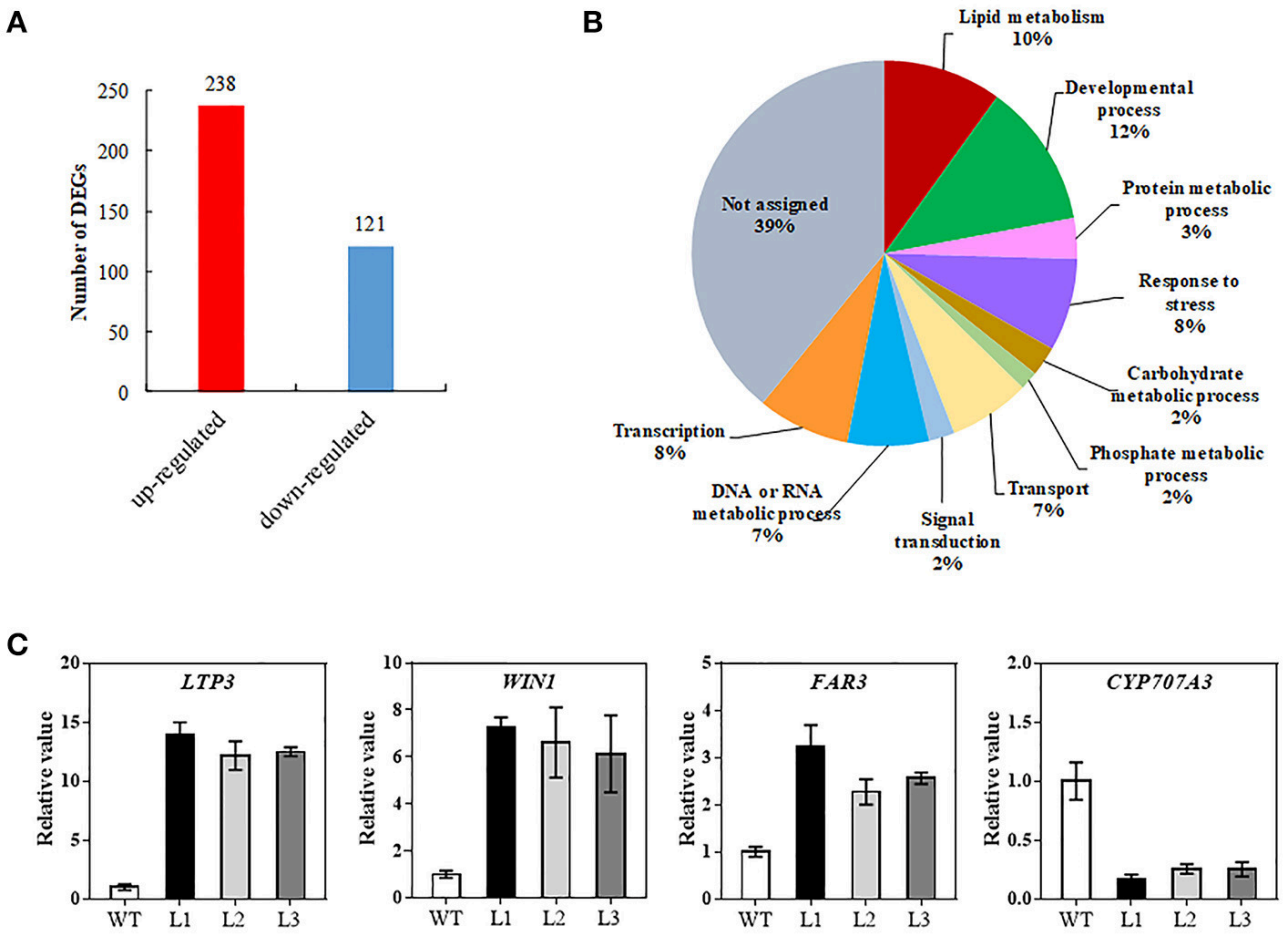
Transcriptome analysis of the *35S::TaMYB31-B* transgenic *Arabidopsis* under drought conditions. **(A)** Number of up- or down-regulated genes in transgenic plants compared with WT plants using a significant cutoff of false discovery rate (FDR) < 0.01, and a fold change (FC) > 2. **(B)** Gene ontology classification for differentially expressed genes (DEGs) between WT and *TaMYB31-B* transgenic plants. **(C)** Confirmation of DEGs expression by quantitative RT-PCR analysis. Error bars represent SD of three replicates.

To decipher the possible mechanism of *TaMYB31* in regulating *Arabidopsis* drought tolerance, comparative gene expression analysis was conducted in transgenic and WT plants using RNA-seq. A total of 359 genes were found to be differentially expressed (fold change >2, false discovery rate <0.01). Of these, 238 genes were up-regulated and 121 were down-regulated in transgenic plants (Figure 8A; Table S2). To obtain the functional information of these genes, we searched for gene ontology (GO) terms using agriGO web-based tool and database. Most of the differentially expressed genes were associated with developmental process, lipid metabolism, and responses to stress (Figure 8B). In particular, a group of genes associated with wax biosynthesis in plants, including *WIN1/SHN1, CYP96A15, FAR3, CER1-L1*, and *WSD1* (Table 1) (Broun et al., 2004; Rowland et al., 2006; Greer et al., 2007; Li et al., 2008; Wang et al., 2018), and those that are involved in water stress responses, including *LTP3, WIN1/SHN1, KIN1*, and *LEA* were up-regulated in transgenic plants (Table 1). Interestingly, *CYP707A3* was found to be down-regulated in transgenic plants, which is involved in the abscisic acid degradation pathway.

**Table 1:** The list of DEGs in *35S::TaMYB31-B* transgenic *Arabidopsis* that are highlighted in this paper.

**Gene ID**	**Gene symbol**	**log2FC**	**Profile**	**Description**	**Function**
AT5G59310	LTP4	5.29	UP	Non-specific lipid-transfer protein 4	lipid metabolism, drought/ABA responses
AT2G21490	LEA	4.87	UP	Probable dehydrin LEA	drought/ABA responses
AT5G59320	LTP3	4.60	UP	Non-specific lipid-transfer protein 3	lipid metabolism, drought/ABA responses
AT4G33355	LTP11	3.84	UP	Non-specific lipid-transfer protein 11	lipid metabolism
AT1G57750	CYP96A15	3.35	UP	Alkane hydroxylase MAH1	wax biosynthesis
AT1G15360	WIN1/SHN1	2.84	UP	WAX INDUCER1/SHINE1	wax biosynthesis, drought/ABA responses
AT4G33790	FAR3	1.81	UP	Fatty acyl-CoA reductase 3	wax biosynthesis
AT3G51590	LTP12	1.70	UP	Non-specific lipid-transfer protein 12	lipid metabolism
AT5G37300	WSD1	1.64	UP	O-acyltransferase WSD1	wax biosynthesis
AT4G12470	AZI1	1.60	UP	pEARLI1-like lipid transfer protein 1	lipid metabolism
AT1G02190	CER1-L1	1.28	UP	CER1-like 1	wax biosynthesis
AT5G15960	KIN1	1.06	UP	Stress-induced protein KIN1	drought/ABA responses
AT2G44080	ARL	−1.17	DOWN	ARGOS-like protein	regulates lateral organ size
AT1G73330	ATDR4	−1.24	DOWN	drought-repressed 4	drought response
AT5G45340	CYP707A3	−1.88	DOWN	Abscisic acid 8'-hydroxylase 3	drought/ABA responses

Several wax biosynthesis and stress-related genes differentially expressed in our transcriptome data were further validated using quantitative RT-PCR. As shown in Figure 8C, the expression levels of *LTP3, WIN1*, and *FAR3* in the transgenic lines were significantly higher than that in control; in contrast, the expression levels of *CYP707A3* in the transgenic lines were significantly lower than that in control. The fold changes in gene expression determined by quantitative RT-PCR were in accordance with the fold changes of normalized expression level (FPKM) by RNA-Seq.

## Discussion

Common wheat (*Triticum aestivum*, BBAADD) is an allohexaploid species originating from two independent hybridization events involving three diploid species, *Triticum urartu* (AA), *Aegilops speltoides* (SS≈BB), and *Aegilops tauschii* (DD) (Gill and Friebe, 2002). Thus, there are generally three homoeologous genes present in common wheat; a large quantity of these homoeologs exhibit expression partitioning (Liu et al., 2015). In the course of evolution, the triplicate homoeologs have three possible fates: preservation of original or similar function, functional diversification, and gene silencing (Wendel, 2000). Functional diversification of homoeologous genes is one of the driving factors leading to the successful evolution of polyploidy species (Shitsukawa et al., 2007). In the present study, the three *TaMYB31s* were differentially expressed; *TaMYB31-B* displayed the highest transcript level in almost all of the tissues examined, especially in flowering spikes, while *TaMYB31-A* and *TaMYB31-D* were less expressed, especially in wheat roots (Figure 2A). Temporal and spatial gene expression is generally associated with specific developmental and physiological functions. Our results suggested that *TaMYB31-B* may play a role in the development of flowering spikes. In addition, we found that *TaMYB31-A* has a rapid response to ABA treatment. These results indicated that functional diversification might have occurred in *TaMYB31s*. Future investigation should focus on analyzing the promoters of the three *TaMYB31* genes to unravel the regulatory mechanism underlying their specific expression patterns.

Many MYBs participate in various stress-related responses in plants. Though more than 84 *MYB* genes have been found in wheat, only a few of them had been functionally analyzed, such as *TaMYB1* (Lee et al., 2007), *TaMYB2A* (Mao et al., 2011), *TaMYB19-B* (Zhang et al., 2014), *TaMYB30-B* (Zhang et al., 2012), *TaMYB33* (Qin et al., 2012), *TaMYB74* (Bi et al., 2016), and *TaMYB80* (Zhao et al., 2017b). In this study, we found that *TaMYB31* genes were up-regulated in wheat leaves by PEG and ABA treatments (Figures 2B,C), implying a potential role of *TaMYB31* genes in stress responses. Indeed, *TaMYB31-B* transgenic *Arabidopsis* plants exhibited enhanced tolerance to drought stress in soil as well as to dehydration stress simulated by PEG treatment (Figures 5, 6).

To reveal possible regulatory mechanisms of *TaMYB31-B* in response to drought stress, transcriptome analysis of WT and L2 was conducted using RNA-Seq. The results showed that the genes involved in lipid metabolism were up-regulated in *TaMYB30-B* overexpressing lines under drought treatment, such as *lipid-transfer protein 3* (*LTP3*), *LTP4, LTP11*, and *LTP12* (Table 1). Plant LTPs participate in the transportation of cuticular waxes during the assembly of plant cuticle (Yeats and Rose, 2008). *Arabidopsis LTP3* was shown to be the direct target of the AtMYB96; overexpression of *LTP3* resulted in a constitutively enhanced tolerance to drought and freezing stresses (Guo et al., 2013). The authors speculated that LTP3 might bind and deliver lipids from cytosol to the cell wall to synthesize cuticular waxes, which protect plants against biotic and abiotic stresses, including drought. In addition, the expression levels of several wax biosynthesis genes, including *WIN1/SHN1, CYP96A15, FAR3, CER1-L1*, and *WSD1*, were up-regulated in *TaMYB31-B* overexpressing plants under drought treatments (Table 1). *Arabidopsis WIN1/SHN1* was the first TF whose role in the regulation of cuticle biosynthesis was experimentally demonstrated; overexpression of *WIN1/SHN1* genes activates cuticular wax biosynthesis, reduces water loss and increases drought tolerance in transgenic Arabidopsis (Aharoni et al., 2004), rice (Wang et al., 2012) and wheat (Bi et al., 2018). The above results suggested that TaMYB31 shared similar functions with its homolog AtMYB96. It was shown that overexpression of the ABA- and drought-inducible *AtMYB96* activates the expression of wax biosynthesis genes and increases the deposition of cuticular waxes, which consequently leads to improved drought tolerance in *Arabidopsis* (Seo et al., 2009, 2011).

It is noteworthy that overexpression of *TaMYB31-B* causes severe morphological defects such as dwarfed stature under normal conditions. Previous research showed that *ARGOS-LIKE* (*AtARL*) regulates lateral organ size (Hu et al., 2006). Increased *AtARL* expression was shown to enlarge organ size, while reduced *AtARL* expression led to organ reduction. We found that *AtARL* was down-regulated in *TaMYB31-B* transgenic plants (Table 1), suggesting that dwarfed stature observed here might result from the deceased expression of *AtARL* by *TaMYB31-B*. In addition, ABA regulates many developmental processes and mediates adaptive responses to environmental stresses. Among others, ABA plays a negative role in seed germination and seedling growth (Koornneef et al., 2002), while promoting tolerance to abiotic stresses including drought (Fujita et al., 2011). It is commonly accepted that the balance between ABA biosynthesis and catabolism determines its action (Seiler et al., 2011). In this study, the expression levels of *TaMYB31s* were increased in response to ABA treatment. Moreover, overexpression of the *TaMYB31-B* gene increased ABA sensitivity in seed germination. RNA-seq data revealed that the expression of *CYP707A3*, a cytochrome P450 CYP707A family gene involved in ABA catabolism and determining threshold levels of ABA during dehydration-rehydration response (Umezawa et al., 2006), is markedly reduced in *TaMYB31-B* transgenic plants (Table 1). Taken together, our results suggest that the growth retardation of *TaMYB31-B*, as well as the improved tolerance to drought are at least partly attributed to ABA.

Although a variety of transgenic crops with drought tolerance have been generated, many of these transgenics show growth retardation (Todaka et al., 2015). Growth retardation is commonly present in transgenic plants which tremendously limits the potential utilization of candidate genes in crop breeding (Mao et al., 2011). To reduce such negative effects, stress inducible promoters have been utilized to subtly control the expression of target genes in transgenic plants (Pino et al., 2007). Recently, it has been reported that application of a gene stacking approach with two transcription factor genes *DREB1A* and *OsPIL1* overcomes growth retardation in drought-tolerant transgenic plants (Kudo et al., 2017). In the present study, *TaMYB31-B* overexpression plants in *Arabidopsis* also showed growth retardation under normal growth conditions. This inspires us to use stress-inducible promoters or the gene stacking approach when utilization of *TaMYB31-B* in crop drought improvement.

## Author contributions

YZ carried out the experiments, analyzed the results, and drafted the manuscript. XC and HX provided the idea and instructed the research work. XL and HW provided assistance to perform experiments and collect data. HB designed the experiments and revised the manuscript. All authors have read and approved the final manuscript.

### Conflict of interest statement

The authors declare that the research was conducted in the absence of any commercial or financial relationships that could be construed as a potential conflict of interest.

## Footnotes

^1^http://www.wheatgenome.org/

^2^https://www.lynnon.com/dnaman.html

^3^http://www.bio-rad.com/
