# Supplementary material for: The Wheat MYB Transcription Factor TaMYB31 Is Involved in Drought Stress Responses in Arabidopsis
Source: Front Plant Sci. 2018 Sep 28;9:1426. doi: 10.3389/fpls.2018.01426 (PMC6172359; doi:10.3389/fpls.2018.01426)
Supplement: Supplementary file 1 [file Data_Sheet_1.DOC]

Supplementary Material

# The wheat MYB transcription factor TaMYB31 is involved in drought stress responses in *Arabidopsis*

Yue Zhao^1^, Xiyong Cheng^2^, Xiaodan Liu^2^, Huifang Wu^2^, Huihui Bi^2^*, Haixia Xu^2^

*Correspondence:

Dr. Huihui Bi

bihuihui826@126.com

# Supplementary Tables

**Table S1.** Primer sequences used in this study.

**Table S2.** List of genes up-regulated or down-regulated (log2 fold change (log2FC) > 1 or <-1, false discovery rate (FDR ) < 0.01) in *TaMYB31-B* transgenic *Arabidopsis* plants relative to WT plants.

**Table S1.** Primer sequences used in this study.

| **Purposes** | **Primer name** | **Sequence information** | **Enzyme site** | |
| --- | --- | --- | --- | --- |
| Gene cloning | TaMYB31-GF | GCCTAGCCAGCCAAGAAGAT |  | |
|  | TaMYB31-GR | CGCCGTACTTAGAAGAACTCAC |  | |
| ORF cloning | TaMYB31-cF | GCCTAGCCAGCCAAGAAGAT |  | |
|  | TaMYB31-cR | CGCCGTACTTAGAAGAACTCAC |  | |
| qRT-PCR | TaMYB31-qAF | CAACCGGGAATTGATCGACT |  | |
|  | TaMYB31-qAR | CATCGCAAAGAGCAACTAATTG |  | |
|  | TaMYB31-qBF | CATGCTGGAGAACTGGCTG |  | |
|  | TaMYB31-qBR | AACAGCGGAAAGAGCAACTAC |  | |
|  | TaMYB31-qDF | CATGCTGGAGAACTGGCTT |  | |
|  | TaMYB31-qDR | TGTTCTTGCTCTCGTCGATG |  | |
|  | β-action-F | GGAATCCATGAGACCACCTAC |  | |
|  | β-action-R | GACCCAGACAACTCGCAAC |  | |
| Subcellular localization | TaMYB31-SF | AGTACCCGGGATGGGGAGGCCTCCGT | *Sma* I | |
|  | TaMYB31-SR | CCATCTGCAGGAAGAACTCACTGGG | *Pst* I |  |
| Transgenic | TaMYB31-TBF | GGGGACAAGTTTGTACAAAAAAGCAGGCTTCATGGGGAGGCCTCCGTG | attB site | |
| *Arabidopsis* | TaMYB31-TBR | GGGGACCACTTTGTACAAGAAAGCTGGGTCTTAGAAGAACTCACTGGGGT | attB site | |
| RNA-seq | LTP3-F | TGGCTCCATGTGCAACCTAT |  | |
| analysis | LTP3-R | GGACTGGATGCATCTGCAAG |  | |
|  | WIN1-F | CTTCATCGCTCTCTTCCATCC |  | |
|  | WIN1-R | CCAATACTTCTTCTCTGCTGC |  | |
|  | FAR3-F | ACCGTGGACCAACAAAGAAG |  | |
|  | FAR3-R | GCAATCAAGTAGCGTATGGTCA |  | |
|  | CYP707A3-F | ATGGATTTCTCCGGTTTGTTTC |  | |
|  | CYP707A3-R | CTATGGTTTTCGTTCCAAGG |  | |
|  | Actin2-F | GCTCCTCTTAACCCAAAGGC |  | |
|  | Actin2-R | CACACCATCACCAGAATCCAGC |  | |

**Table S2.** List of genes up-regulated or down-regulated (log2 fold change (log2FC) > 1 or <-1, false discovery rate (FDR ) < 0.01) in *TaMYB31-B* transgenic *Arabidopsis* plants relative to WT plants.

| **Gene ID** | **log2FC** | **P-value** | **FDR** | **Profile** | **Gene name** |
| --- | --- | --- | --- | --- | --- |
| AT3G25050 | 9.78 | 3.82E-27 | 8.49E-25 | UP | XTH3 |
| AT2G07560 | 9.65 | 1.71E-38 | 7.79E-36 | UP | AHA6 |
| AT1G23240 | 8.38 | 1.65E-18 | 1.71E-16 | UP | PXG7 |
| AT5G61720 | 8.32 | 1.23E-12 | 6.25E-11 | UP |  |
| AT1G29140 | 8.00 | 3.39E-16 | 2.77E-14 | UP |  |
| AT5G07540 | 7.89 | 4.18E-13 | 2.28E-11 | UP | GRP16 |
| AT4G11760 | 7.79 | 1.94E-13 | 1.11E-11 | UP | LCR17 |
| AT1G72290 | 7.75 | 4.11E-14 | 2.58E-12 | UP |  |
| AT3G28980 | 7.41 | 3.95E-64 | 6.14E-61 | UP |  |
| AT1G71680 | 7.23 | 7.82E-10 | 2.60E-08 | UP |  |
| AT1G72110 | 6.89 | 7.86E-09 | 2.18E-07 | UP |  |
| AT5G44300 | 6.84 | 6.37E-08 | 1.48E-06 | UP |  |
| AT3G14040 | 6.62 | 7.06E-76 | 1.88E-72 | UP |  |
| AT3G28820 | 6.43 | 1.10E-06 | 2.00E-05 | UP |  |
| AT1G54860 | 6.10 | 9.90E-06 | 1.40E-04 | UP |  |
| AT1G22760 | 6.05 | 1.28E-20 | 1.63E-18 | UP | PAB3 |
| AT3G28790 | 5.97 | 1.82E-83 | 5.66E-80 | UP |  |
| AT3G28780 | 5.68 | 1.49E-15 | 1.14E-13 | UP |  |
| AT1G69940 | 5.53 | 1.04E-31 | 2.97E-29 | UP | PPME1 |
| AT2G23800 | 5.39 | 9.74E-14 | 5.89E-12 | UP | GGPPS2 |
| AT5G59310 | 5.29 | 4.15E-135 | 3.87E-131 | UP | LTP4 |
| AT3G28830 | 5.02 | 2.55E-48 | 2.06E-45 | UP |  |
| AT5G09550 | 4.90 | 8.22E-10 | 2.72E-08 | UP |  |
| AT2G21490 | 4.87 | 9.10E-10 | 2.97E-08 | UP | LEA |
| AT3G07850 | 4.84 | 5.03E-54 | 5.21E-51 | UP |  |
| AT3G08560 | 4.76 | 1.33E-08 | 3.58E-07 | UP | VHA-E2 |
| AT1G80660 | 4.75 | 6.96E-24 | 1.19E-21 | UP | AHA9 |
| AT4G12960 | 4.75 | 5.89E-10 | 2.02E-08 | UP | GILT |
| AT1G28430 | 4.67 | 1.52E-10 | 5.68E-09 | UP | CYP705A24 |
| AT5G07410 | 4.64 | 1.08E-58 | 1.18E-55 | UP | PME48 |
| AT3G62170 | 4.62 | 2.22E-30 | 6.01E-28 | UP | VGDH2 |
| AT5G59320 | 4.60 | 2.60E-165 | 4.85E-161 | UP | LTP3 |
| AT1G55570 | 4.55 | 4.82E-20 | 5.76E-18 | UP | sks12 |
| AT3G19090 | 4.51 | 1.60E-07 | 3.41E-06 | UP | LARP6C |
| AT1G02790 | 4.44 | 2.30E-36 | 8.42E-34 | UP | PGA3 |
| AT3G05960 | 4.43 | 3.16E-07 | 6.35E-06 | UP | STP6 |
| AT1G18280 | 4.24 | 6.57E-10 | 2.23E-08 | UP |  |
| AT2G19800 | 4.14 | 5.55E-06 | 8.39E-05 | UP | MIOX2 |
| AT5G07530 | 4.12 | 1.89E-61 | 2.52E-58 | UP | GRP17 |
| AT3G59930 | 4.03 | 1.15E-05 | 1.61E-04 | UP |  |
| AT1G48470 | 4.03 | 1.44E-05 | 1.97E-04 | UP | GLN1-5 |
| AT5G36910 | 3.96 | 2.15E-08 | 5.55E-07 | UP | THI2 |
| AT5G07550 | 3.94 | 3.94E-35 | 1.41E-32 | UP | GRP19 |
| AT3G57690 | 3.90 | 3.15E-05 | 3.88E-04 | UP | AGP23 |
| AT5G54190 | 3.85 | 5.00E-38 | 2.12E-35 | UP | PORA |
| AT4G33355 | 3.84 | 3.96E-13 | 2.18E-11 | UP | LTP11 |
| AT5G53820 | 3.84 | 1.82E-12 | 9.02E-11 | UP |  |
| AT3G42850 | 3.81 | 1.28E-06 | 2.29E-05 | UP |  |
| AT3G01270 | 3.79 | 3.74E-26 | 7.34E-24 | UP |  |
| AT2G39510 | 3.72 | 1.79E-06 | 3.09E-05 | UP |  |
| AT5G07510 | 3.65 | 4.76E-08 | 1.13E-06 | UP | GRP14 |
| AT5G45880 | 3.56 | 4.32E-07 | 8.49E-06 | UP |  |
| AT3G58590 | 3.56 | 1.61E-74 | 3.74E-71 | UP |  |
| AT4G25850 | 3.56 | 2.98E-10 | 1.09E-08 | UP | ORP4B |
| AT2G47040 | 3.47 | 2.12E-38 | 9.41E-36 | UP | PME5 |
| AT2G47050 | 3.44 | 2.90E-18 | 2.95E-16 | UP |  |
| AT3G07820 | 3.39 | 7.65E-18 | 7.59E-16 | UP |  |
| AT1G57750 | 3.35 | 6.33E-121 | 3.94E-117 | UP | CYP96A15 |
| AT3G46520 | 3.28 | 3.97E-05 | 4.76E-04 | UP | ACT12 |
| AT1G23670 | 3.28 | 3.56E-05 | 4.33E-04 | UP |  |
| AT1G75930 | 3.20 | 1.80E-12 | 8.95E-11 | UP | EXL6 |
| AT5G50030 | 3.17 | 1.03E-04 | 1.10E-03 | UP |  |
| AT3G13400 | 3.15 | 2.26E-37 | 8.96E-35 | UP | sks13 |
| AT1G68170 | 3.11 | 1.45E-04 | 1.48E-03 | UP |  |
| AT4G08670 | 3.11 | 4.99E-11 | 2.00E-09 | UP |  |
| AT1G23570 | 3.09 | 4.17E-08 | 1.01E-06 | UP |  |
| AT5G07560 | 3.08 | 8.25E-17 | 7.23E-15 | UP | GRP20 |
| AT5G60510 | 3.04 | 1.55E-05 | 2.10E-04 | UP |  |
| AT1G75910 | 3.00 | 1.08E-19 | 1.24E-17 | UP | EXL4 |
| AT1G23580 | 3.00 | 5.13E-04 | 4.37E-03 | UP |  |
| AT4G38410 | 2.95 | 1.59E-07 | 3.39E-06 | UP |  |
| AT5G20710 | 2.93 | 6.35E-16 | 5.02E-14 | UP | BGAL7 |
| AT3G49540 | 2.92 | 6.28E-04 | 5.17E-03 | UP |  |
| AT3G18360 | 2.88 | 1.87E-05 | 2.49E-04 | UP | VQ20 |
| AT1G35290 | 2.87 | 2.44E-07 | 5.02E-06 | UP |  |
| AT1G15360 | 2.84 | 1.74E-08 | 4.60E-07 | UP | WIN1 |
| AT3G21180 | 2.80 | 1.14E-04 | 1.21E-03 | UP | ACA9 |
| AT4G36350 | 2.79 | 1.43E-05 | 1.96E-04 | UP | PAP25 |
| AT3G13390 | 2.77 | 4.40E-08 | 1.06E-06 | UP | sks11 |
| AT3G02480 | 2.77 | 2.68E-09 | 8.02E-08 | UP |  |
| AT4G35010 | 2.72 | 4.96E-10 | 1.72E-08 | UP | BGAL11 |
| AT5G37970 | 2.66 | 4.63E-05 | 5.46E-04 | UP |  |
| AT5G51950 | 2.63 | 1.01E-16 | 8.80E-15 | UP |  |
| AT3G13433 | 2.61 | 4.64E-04 | 4.01E-03 | UP |  |
| AT1G26720 | 2.61 | 2.45E-04 | 2.30E-03 | UP |  |
| AT4G29340 | 2.54 | 2.39E-04 | 2.26E-03 | UP | PRO3 |
| AT2G33880 | 2.45 | 2.70E-06 | 4.43E-05 | UP | WOX9 |
| AT1G80160 | 2.44 | 1.31E-04 | 1.37E-03 | UP |  |
| AT3G57380 | 2.35 | 6.62E-04 | 5.42E-03 | UP |  |
| AT1G75940 | 2.32 | 2.29E-16 | 1.89E-14 | UP | BGLU20 |
| AT1G15460 | 2.28 | 2.64E-06 | 4.34E-05 | UP | BOR4 |
| AT1G02070 | 2.23 | 1.34E-03 | 9.86E-03 | UP |  |
| AT1G66850 | 2.19 | 1.11E-09 | 3.56E-08 | UP |  |
| AT2G30770 | 2.18 | 4.72E-38 | 2.05E-35 | UP | CYP71A13 |
| AT1G23560 | 2.17 | 2.95E-06 | 4.81E-05 | UP |  |
| AT4G04223 | 2.16 | 7.54E-09 | 2.10E-07 | UP |  |
| AT4G14695 | 2.14 | 1.40E-04 | 1.44E-03 | UP | MPC2 |
| AT2G03850 | 2.14 | 4.94E-04 | 4.22E-03 | UP |  |
| AT1G24260 | 2.10 | 2.67E-18 | 2.74E-16 | UP | 3-Sep |
| AT5G57785 | 2.08 | 4.83E-06 | 7.40E-05 | UP |  |
| AT1G68875 | 2.06 | 5.21E-09 | 1.49E-07 | UP |  |
| AT5G50790 | 2.05 | 4.79E-11 | 1.94E-09 | UP | SWEET10 |
| AT5G24080 | 2.04 | 5.04E-05 | 5.87E-04 | UP |  |
| AT5G07430 | 2.00 | 5.31E-06 | 8.07E-05 | UP | PME50 |
| AT3G15400 | 1.99 | 7.18E-17 | 6.35E-15 | UP | ATA20 |
| AT2G39518 | 1.98 | 2.72E-04 | 2.51E-03 | UP |  |
| AT1G06350 | 1.96 | 1.59E-29 | 4.02E-27 | UP |  |
| AT1G52855 | 1.91 | 1.17E-09 | 3.74E-08 | UP |  |
| AT3G02310 | 1.91 | 2.33E-09 | 7.05E-08 | UP | 2-Sep |
| AT3G01700 | 1.91 | 2.83E-05 | 3.55E-04 | UP | AGP11 |
| AT3G24340 | 1.91 | 3.84E-04 | 3.40E-03 | UP | CLSY4 |
| AT5G14380 | 1.91 | 6.68E-06 | 9.91E-05 | UP | AGP6 |
| AT5G48210 | 1.89 | 3.87E-05 | 4.66E-04 | UP |  |
| AT3G11340 | 1.88 | 1.37E-04 | 1.42E-03 | UP |  |
| AT3G28750 | 1.84 | 1.56E-08 | 4.16E-07 | UP |  |
| AT4G30660 | 1.82 | 2.63E-05 | 3.33E-04 | UP |  |
| AT4G33790 | 1.81 | 1.03E-25 | 1.95E-23 | UP | FAR3 |
| AT2G14247 | 1.78 | 1.69E-04 | 1.68E-03 | UP |  |
| AT5G49120 | 1.78 | 9.16E-05 | 9.96E-04 | UP |  |
| AT5G37940 | 1.74 | 5.60E-34 | 1.83E-31 | UP |  |
| AT4G04460 | 1.74 | 2.07E-13 | 1.18E-11 | UP | APA3 |
| AT5G10440 | 1.71 | 7.34E-04 | 5.87E-03 | UP | CYCD4-2 |
| AT5G22430 | 1.71 | 1.66E-48 | 1.41E-45 | UP |  |
| AT1G50310 | 1.71 | 4.65E-08 | 1.11E-06 | UP | STP9 |
| AT1G20120 | 1.70 | 2.40E-06 | 3.99E-05 | UP |  |
| AT3G51590 | 1.70 | 4.04E-15 | 2.90E-13 | UP | LTP12 |
| AT4G12500 | 1.69 | 2.09E-13 | 1.19E-11 | UP |  |
| AT4G27460 | 1.69 | 1.91E-04 | 1.86E-03 | UP | CBSX5 |
| AT4G14630 | 1.67 | 2.76E-04 | 2.54E-03 | UP | GLP9 |
| AT3G19390 | 1.67 | 5.76E-04 | 4.83E-03 | UP | AT3G19400 |
| AT1G58430 | 1.66 | 3.25E-06 | 5.23E-05 | UP | RXF26 |
| AT2G13550 | 1.65 | 4.99E-05 | 5.83E-04 | UP |  |
| AT1G20130 | 1.64 | 3.29E-09 | 9.70E-08 | UP | APG |
| AT1G71770 | 1.64 | 2.22E-06 | 3.75E-05 | UP | PAB5 |
| AT5G37300 | 1.64 | 3.82E-16 | 3.08E-14 | UP | WSD1 |
| AT1G65730 | 1.62 | 1.34E-04 | 1.39E-03 | UP | YSL7 |
| AT2G20870 | 1.62 | 5.81E-45 | 3.87E-42 | UP |  |
| ATCG00090 | 1.62 | 5.66E-14 | 3.50E-12 | UP |  |
| AT4G15670 | 1.62 | 2.31E-04 | 2.19E-03 | UP | GRXS7 |
| AT4G27570 | 1.61 | 9.87E-04 | 7.56E-03 | UP | UGT79B3 |
| AT2G05540 | 1.61 | 3.36E-12 | 1.60E-10 | UP |  |
| AT4G12470 | 1.60 | 2.58E-13 | 1.45E-11 | UP | AZI1 |
| AT1G53160 | 1.59 | 2.17E-07 | 4.50E-06 | UP | SPL4 |
| AT3G25717 | 1.58 | 1.20E-04 | 1.26E-03 | UP | RTFL16 |
| AT4G30180 | 1.58 | 7.28E-04 | 5.83E-03 | UP |  |
| AT5G16960 | 1.57 | 4.47E-06 | 6.91E-05 | UP |  |
| AT2G19070 | 1.57 | 1.73E-04 | 1.71E-03 | UP | SHT |
| AT5G21150 | 1.56 | 3.16E-09 | 9.36E-08 | UP | AGO9 |
| AT5G44630 | 1.56 | 1.98E-04 | 1.91E-03 | UP | BS |
| AT3G14060 | 1.54 | 1.98E-07 | 4.15E-06 | UP |  |
| AT2G03740 | 1.53 | 1.07E-03 | 8.07E-03 | UP |  |
| AT4G01895 | 1.52 | 2.41E-04 | 2.27E-03 | UP |  |
| AT4G24000 | 1.52 | 5.21E-09 | 1.49E-07 | UP | CSLG2 |
| AT4G08770 | 1.51 | 6.42E-06 | 9.57E-05 | UP | PER37 |
| AT5G14410 | 1.50 | 3.58E-05 | 4.35E-04 | UP |  |
| AT5G39860 | 1.49 | 1.26E-06 | 2.27E-05 | UP | PRE1 |
| AT1G61110 | 1.48 | 5.54E-04 | 4.67E-03 | UP | NAC025 |
| AT2G31980 | 1.48 | 1.50E-04 | 1.52E-03 | UP | CYS2 |
| AT4G22070 | 1.47 | 1.41E-04 | 1.45E-03 | UP | WRKY31 |
| AT5G51910 | 1.47 | 2.74E-05 | 3.45E-04 | UP | TCP19 |
| AT5G50360 | 1.45 | 3.21E-04 | 2.91E-03 | UP |  |
| AT5G19580 | 1.44 | 4.01E-13 | 2.20E-11 | UP |  |
| AT1G67260 | 1.44 | 8.67E-06 | 1.24E-04 | UP | TCP1 |
| AT5G55720 | 1.43 | 4.77E-04 | 4.10E-03 | UP |  |
| AT4G14746 | 1.42 | 3.91E-14 | 2.46E-12 | UP |  |
| AT1G69120 | 1.41 | 1.97E-12 | 9.73E-11 | UP | AP1 |
| AT3G28840 | 1.41 | 4.67E-04 | 4.03E-03 | UP |  |
| AT4G14815 | 1.41 | 5.86E-04 | 4.89E-03 | UP |  |
| AT3G22600 | 1.40 | 3.68E-15 | 2.66E-13 | UP |  |
| AT5G47330 | 1.40 | 3.86E-12 | 1.82E-10 | UP |  |
| AT1G78950 | 1.39 | 2.05E-04 | 1.98E-03 | UP | BAS |
| AT5G45960 | 1.39 | 5.33E-06 | 8.11E-05 | UP |  |
| AT1G35230 | 1.39 | 1.37E-13 | 8.08E-12 | UP | AGP5 |
| AT4G37990 | 1.38 | 3.77E-08 | 9.25E-07 | UP | CAD8 |
| AT2G25297 | 1.36 | 8.07E-04 | 6.38E-03 | UP |  |
| AT5G03840 | 1.33 | 5.95E-04 | 4.94E-03 | UP | TFL1 |
| AT4G01430 | 1.33 | 4.71E-04 | 4.05E-03 | UP |  |
| AT5G15800 | 1.33 | 9.11E-11 | 3.51E-09 | UP | 1-Sep |
| AT1G34640 | 1.32 | 6.90E-04 | 5.60E-03 | UP |  |
| AT4G35905 | 1.31 | 1.66E-04 | 1.65E-03 | UP |  |
| AT3G46020 | 1.30 | 2.09E-04 | 2.01E-03 | UP |  |
| AT1G17920 | 1.30 | 1.92E-06 | 3.28E-05 | UP | HDG12 |
| AT3G54340 | 1.30 | 2.23E-06 | 3.76E-05 | UP | AP3 |
| AT5G20240 | 1.30 | 2.01E-06 | 3.42E-05 | UP | PI |
| AT5G37950 | 1.29 | 2.21E-04 | 2.11E-03 | UP |  |
| AT1G66810 | 1.29 | 1.24E-03 | 9.18E-03 | UP |  |
| AT5G13330 | 1.29 | 5.30E-04 | 4.49E-03 | UP | ERF113 |
| AT5G39220 | 1.29 | 5.25E-04 | 4.45E-03 | UP |  |
| AT1G52827 | 1.28 | 3.23E-04 | 2.92E-03 | UP | CDT1 |
| AT3G09270 | 1.28 | 3.63E-04 | 3.23E-03 | UP | GSTU8 |
| AT1G02190 | 1.28 | 5.60E-13 | 2.97E-11 | UP | CER1-L1 |
| AT3G20440 | 1.26 | 3.61E-13 | 2.00E-11 | UP | SBE3 |
| AT4G08150 | 1.25 | 2.20E-14 | 1.43E-12 | UP | KNAT1 |
| AT1G78960 | 1.24 | 6.05E-10 | 2.07E-08 | UP | LUP2 |
| AT5G13380 | 1.23 | 7.25E-05 | 8.12E-04 | UP |  |
| AT3G07590 | 1.23 | 4.55E-04 | 3.95E-03 | UP |  |
| AT2G36050 | 1.22 | 5.51E-05 | 6.35E-04 | UP | OFP15 |
| AT1G67990 | 1.22 | 1.26E-05 | 1.75E-04 | UP | TSM1 |
| AT1G08630 | 1.22 | 3.93E-04 | 3.47E-03 | UP | THA1 |
| AT5G20635 | 1.22 | 1.24E-05 | 1.72E-04 | UP | GG3 |
| AT5G23480 | 1.21 | 5.85E-04 | 4.88E-03 | UP |  |
| AT4G21590 | 1.20 | 3.04E-04 | 2.78E-03 | UP | ENDO3 |
| AT1G74240 | 1.17 | 1.93E-04 | 1.88E-03 | UP |  |
| AT3G59510 | 1.17 | 1.35E-04 | 1.40E-03 | UP |  |
| AT1G33960 | 1.17 | 6.60E-06 | 9.80E-05 | UP | AIG1 |
| AT5G38900 | 1.16 | 1.13E-06 | 2.05E-05 | UP |  |
| AT2G26400 | 1.15 | 1.36E-04 | 1.41E-03 | UP | ARD1 |
| AT1G76930 | 1.15 | 1.04E-31 | 2.97E-29 | UP | ATEXT4 |
| AT3G15450 | 1.14 | 4.03E-32 | 1.21E-29 | UP |  |
| AT2G45220 | 1.14 | 4.64E-15 | 3.29E-13 | UP | PME17 |
| AT3G11000 | 1.14 | 4.47E-05 | 5.30E-04 | UP |  |
| AT2G27880 | 1.12 | 1.13E-03 | 8.48E-03 | UP | AGO5 |
| AT5G66330 | 1.11 | 3.39E-04 | 3.05E-03 | UP |  |
| AT5G15010 | 1.11 | 1.17E-03 | 8.77E-03 | UP |  |
| AT4G23680 | 1.09 | 2.27E-08 | 5.81E-07 | UP |  |
| AT2G35310 | 1.09 | 9.37E-04 | 7.24E-03 | UP |  |
| AT2G20750 | 1.09 | 3.29E-07 | 6.57E-06 | UP | EXPB1 |
| AT4G00870 | 1.08 | 2.81E-05 | 3.52E-04 | UP | BHLH14 |
| AT3G09480 | 1.07 | 3.84E-04 | 3.40E-03 | UP |  |
| AT2G35980 | 1.07 | 3.79E-05 | 4.57E-04 | UP | YLS9 |
| AT1G05340 | 1.06 | 2.94E-06 | 4.80E-05 | UP |  |
| AT5G15960 | 1.06 | 2.96E-08 | 7.43E-07 | UP | KIN1 |
| AT1G67590 | 1.05 | 5.02E-08 | 1.19E-06 | UP |  |
| AT3G22840 | 1.05 | 9.53E-06 | 1.36E-04 | UP | ELIP1 |
| AT1G23390 | 1.05 | 6.88E-21 | 9.16E-19 | UP |  |
| AT4G28190 | 1.05 | 1.14E-03 | 8.53E-03 | UP | ULT1 |
| AT1G46264 | 1.05 | 1.38E-06 | 2.45E-05 | UP | HSFB4 |
| AT4G28680 | 1.04 | 2.22E-05 | 2.88E-04 | UP | TYRDC |
| AT5G52900 | 1.03 | 4.14E-05 | 4.93E-04 | UP | MAKR6 |
| AT3G01330 | 1.03 | 4.93E-04 | 4.22E-03 | UP | E2FF |
| AT4G23870 | 1.03 | 1.21E-03 | 9.00E-03 | UP |  |
| AT5G02440 | 1.03 | 1.16E-03 | 8.68E-03 | UP |  |
| AT4G14080 | 1.03 | 1.71E-06 | 2.97E-05 | UP | A6 |
| AT3G01970 | 1.02 | 8.04E-04 | 6.36E-03 | UP | WRKY45 |
| AT1G79110 | 1.01 | 4.10E-06 | 6.40E-05 | UP | BRG2 |
| AT4G19700 | 1.01 | 1.55E-05 | 2.10E-04 | UP | BOI |
| AT2G29460 | 1.01 | 1.08E-07 | 2.39E-06 | UP | GSTU4 |
| AT3G06320 | 1.00 | 9.46E-04 | 7.30E-03 | UP |  |
| ATCG00720 | -1.00 | 2.93E-07 | 5.90E-06 | DOWN | PETB |
| AT4G35090 | -1.00 | 1.29E-62 | 1.85E-59 | DOWN | CAT2 |
| AT1G72840 | -1.01 | 2.27E-04 | 2.16E-03 | DOWN |  |
| AT2G34930 | -1.01 | 3.74E-46 | 2.79E-43 | DOWN |  |
| AT5G59670 | -1.02 | 1.81E-23 | 2.96E-21 | DOWN |  |
| AT2G39310 | -1.02 | 3.41E-09 | 1.01E-07 | DOWN | JAL22 |
| AT4G08950 | -1.02 | 9.95E-21 | 1.29E-18 | DOWN | EXO |
| AT4G11911 | -1.03 | 3.42E-04 | 3.06E-03 | DOWN |  |
| AT1G62660 | -1.03 | 2.23E-29 | 5.46E-27 | DOWN | BFRUCT3 |
| AT4G19520 | -1.03 | 3.17E-45 | 2.19E-42 | DOWN |  |
| AT1G77760 | -1.03 | 8.40E-38 | 3.48E-35 | DOWN | NIA1 |
| AT5G62520 | -1.04 | 3.16E-05 | 3.89E-04 | DOWN | SRO5 |
| AT3G21150 | -1.04 | 1.91E-08 | 5.00E-07 | DOWN | BBX32 |
| AT5G53750 | -1.04 | 1.94E-22 | 2.90E-20 | DOWN |  |
| AT3G50800 | -1.04 | 8.55E-05 | 9.36E-04 | DOWN |  |
| AT5G19100 | -1.04 | 4.50E-04 | 3.91E-03 | DOWN |  |
| AT2G32200 | -1.05 | 6.92E-04 | 5.61E-03 | DOWN |  |
| AT4G29780 | -1.05 | 7.38E-43 | 4.30E-40 | DOWN |  |
| AT3G45140 | -1.06 | 8.31E-65 | 1.55E-61 | DOWN | LOX2 |
| ATCG01070 | -1.06 | 2.33E-07 | 4.82E-06 | DOWN | NDHE |
| AT1G61460 | -1.06 | 2.84E-05 | 3.55E-04 | DOWN |  |
| AT1G33610 | -1.07 | 1.46E-23 | 2.42E-21 | DOWN |  |
| AT3G25570 | -1.08 | 2.82E-09 | 8.39E-08 | DOWN | SAMDC3 |
| AT1G50745 | -1.08 | 7.05E-16 | 5.52E-14 | DOWN |  |
| AT4G13570 | -1.08 | 7.81E-05 | 8.65E-04 | DOWN | HTA4 |
| ATCG01050 | -1.09 | 1.90E-14 | 1.25E-12 | DOWN | NDHD |
| ATCG00500 | -1.09 | 5.14E-07 | 9.99E-06 | DOWN | ACCD |
| AT1G07180 | -1.09 | 5.01E-22 | 7.14E-20 | DOWN | NDA1 |
| AT3G45650 | -1.10 | 3.55E-39 | 1.65E-36 | DOWN | NPF2 |
| AT1G72430 | -1.11 | 3.56E-08 | 8.78E-07 | DOWN |  |
| AT4G24110 | -1.11 | 1.37E-05 | 1.88E-04 | DOWN |  |
| AT2G34655 | -1.11 | 1.22E-07 | 2.68E-06 | DOWN |  |
| AT1G05540 | -1.12 | 1.13E-08 | 3.08E-07 | DOWN |  |
| AT5G21960 | -1.12 | 3.11E-08 | 7.78E-07 | DOWN | ERF016 |
| AT4G13575 | -1.12 | 3.23E-09 | 9.56E-08 | DOWN |  |
| AT3G44990 | -1.12 | 1.49E-09 | 4.67E-08 | DOWN | XTH31 |
| AT3G22060 | -1.12 | 6.54E-27 | 1.42E-24 | DOWN | CRRSP38 |
| AT1G31690 | -1.12 | 1.38E-43 | 8.58E-41 | DOWN |  |
| AT1G52000 | -1.12 | 1.71E-51 | 1.68E-48 | DOWN | JAL5 |
| AT3G46490 | -1.13 | 2.35E-04 | 2.22E-03 | DOWN |  |
| AT4G15550 | -1.14 | 1.59E-14 | 1.04E-12 | DOWN | UGT75D1 |
| AT1G45201 | -1.14 | 9.64E-61 | 1.20E-57 | DOWN | ATTLL1 |
| AT4G25490 | -1.15 | 3.98E-06 | 6.23E-05 | DOWN |  |
| AT3G17690 | -1.16 | 4.76E-13 | 2.56E-11 | DOWN | CNGC19 |
| AT1G52410 | -1.16 | 2.81E-69 | 5.83E-66 | DOWN | TSA1 |
| AT2G35710 | -1.17 | 2.40E-15 | 1.79E-13 | DOWN | PGSIP8 |
| AT2G01021 | -1.17 | 9.63E-07 | 1.76E-05 | DOWN |  |
| AT2G44080 | -1.17 | 7.27E-06 | 1.07E-04 | DOWN | ARL |
| AT2G15020 | -1.17 | 3.75E-22 | 5.38E-20 | DOWN |  |
| AT5G42800 | -1.17 | 2.64E-07 | 5.36E-06 | DOWN | DFRA |
| AT5G44050 | -1.18 | 1.20E-13 | 7.21E-12 | DOWN |  |
| AT3G27250 | -1.18 | 2.31E-06 | 3.90E-05 | DOWN |  |
| ATCG00540 | -1.18 | 5.56E-33 | 1.79E-30 | DOWN | PETA |
| AT4G18440 | -1.19 | 1.09E-64 | 1.84E-61 | DOWN |  |
| AT4G16590 | -1.20 | 1.05E-22 | 1.61E-20 | DOWN | ATCSLA01 |
| AT1G52040 | -1.20 | 5.25E-41 | 2.80E-38 | DOWN | MBP1 |
| ATCG00290 | -1.20 | 2.28E-50 | 2.12E-47 | DOWN |  |
| AT2G27402 | -1.20 | 5.49E-10 | 1.89E-08 | DOWN |  |
| ATCG01060 | -1.21 | 6.99E-08 | 1.61E-06 | DOWN | PSAC |
| AT1G05675 | -1.22 | 1.38E-07 | 2.98E-06 | DOWN | UGT74E1 |
| ATCG00520 | -1.23 | 2.54E-11 | 1.06E-09 | DOWN | YCF4 |
| ATCG00280 | -1.24 | 1.33E-28 | 3.18E-26 | DOWN | PSBC |
| AT5G17350 | -1.24 | 4.05E-05 | 4.84E-04 | DOWN |  |
| AT5G52570 | -1.24 | 2.76E-13 | 1.54E-11 | DOWN | BETA-OHASE |
| AT1G73330 | -1.24 | 1.16E-30 | 3.24E-28 | DOWN | ATDR4 |
| AT5G19110 | -1.24 | 9.10E-26 | 1.77E-23 | DOWN |  |
| AT5G07110 | -1.25 | 9.79E-04 | 7.52E-03 | DOWN | PRA1B6 |
| AT4G30280 | -1.25 | 2.59E-17 | 2.44E-15 | DOWN | XTH18 |
| AT5G44430 | -1.26 | 6.34E-11 | 2.49E-09 | DOWN | PDF1 |
| AT4G19460 | -1.26 | 4.02E-04 | 3.53E-03 | DOWN |  |
| ENSRNA049494871 | -1.27 | 1.38E-04 | 1.42E-03 | DOWN |  |
| AT5G24290 | -1.28 | 1.80E-04 | 1.77E-03 | DOWN | MEB2 |
| AT2G19650 | -1.31 | 2.46E-12 | 1.21E-10 | DOWN |  |
| AT1G52400 | -1.32 | 1.78E-98 | 8.31E-95 | DOWN | BGLU18 |
| AT1G09180 | -1.32 | 5.22E-07 | 1.01E-05 | DOWN | ATSAR1 |
| AT4G17470 | -1.34 | 3.11E-30 | 8.29E-28 | DOWN |  |
| AT2G07777 | -1.36 | 6.82E-04 | 5.55E-03 | DOWN |  |
| AT1G80130 | -1.42 | 2.88E-15 | 2.11E-13 | DOWN |  |
| AT1G11340 | -1.42 | 4.74E-04 | 4.08E-03 | DOWN | RKS1 |
| ATCG00710 | -1.43 | 1.87E-07 | 3.92E-06 | DOWN | PSBH |
| AT2G23010 | -1.43 | 8.70E-10 | 2.86E-08 | DOWN | SCPL9 |
| AT1G62440 | -1.44 | 6.73E-10 | 2.27E-08 | DOWN | LRX2 |
| AT1G56250 | -1.44 | 1.23E-03 | 9.15E-03 | DOWN | VBF |
| AT4G34380 | -1.45 | 3.24E-04 | 2.93E-03 | DOWN |  |
| AT5G61160 | -1.47 | 2.77E-17 | 2.59E-15 | DOWN | ACT |
| AT3G16460 | -1.49 | 3.80E-20 | 4.63E-18 | DOWN | JAL34 |
| AT3G61920 | -1.52 | 1.01E-04 | 1.09E-03 | DOWN |  |
| AT5G38212 | -1.52 | 1.83E-07 | 3.84E-06 | DOWN |  |
| AT3G28220 | -1.53 | 9.12E-50 | 8.10E-47 | DOWN |  |
| ATCG00510 | -1.54 | 2.93E-04 | 2.68E-03 | DOWN | PSAI |
| AT5G61350 | -1.55 | 8.85E-05 | 9.64E-04 | DOWN |  |
| AT4G01080 | -1.56 | 5.26E-24 | 9.17E-22 | DOWN | TBL26 |
| AT2G27420 | -1.58 | 6.44E-08 | 1.50E-06 | DOWN |  |
| AT4G01950 | -1.59 | 6.30E-30 | 1.64E-27 | DOWN | GPAT3 |
| AT2G36120 | -1.60 | 3.09E-32 | 9.59E-30 | DOWN | DOT1 |
| ATCG00040 | -1.60 | 1.44E-36 | 5.36E-34 | DOWN | MATK |
| AT3G52450 | -1.61 | 4.26E-08 | 1.03E-06 | DOWN | PUB22 |
| AT1G33760 | -1.63 | 7.44E-05 | 8.30E-04 | DOWN | ERF022 |
| AT4G22517 | -1.68 | 1.98E-04 | 1.91E-03 | DOWN |  |
| AT3G45130 | -1.72 | 3.27E-04 | 2.95E-03 | DOWN | LAS1 |
| AT1G43800 | -1.73 | 9.87E-12 | 4.39E-10 | DOWN | S-ACP-DES6 |
| AT5G38940 | -1.73 | 7.01E-06 | 1.04E-04 | DOWN |  |
| AT3G16450 | -1.75 | 4.80E-10 | 1.67E-08 | DOWN | JAL33 |
| AT4G01390 | -1.76 | 5.16E-04 | 4.39E-03 | DOWN |  |
| AT1G35140 | -1.76 | 5.83E-08 | 1.37E-06 | DOWN | EXL1 |
| AT5G45340 | -1.88 | 1.33E-26 | 2.79E-24 | DOWN | CYP707A3 |
| AT1G68238 | -1.90 | 1.35E-03 | 9.91E-03 | DOWN |  |
| AT1G73325 | -2.01 | 4.67E-25 | 8.79E-23 | DOWN |  |
| ATCG00950 | -2.03 | 8.57E-08 | 1.94E-06 | DOWN |  |
| AT4G11650 | -2.03 | 5.83E-06 | 8.75E-05 | DOWN | OSM34 |
| AT1G14250 | -2.15 | 1.05E-87 | 3.91E-84 | DOWN | APY5 |
| ENSRNA049494796 | -2.22 | 2.52E-12 | 1.23E-10 | DOWN |  |
| AT4G37410 | -2.24 | 1.85E-04 | 1.81E-03 | DOWN | CYP81F4 |
| AT3G23510 | -2.29 | 4.63E-08 | 1.11E-06 | DOWN |  |
| AT1G30190 | -2.32 | 2.96E-06 | 4.81E-05 | DOWN |  |
| AT1G58400 | -2.51 | 1.17E-04 | 1.23E-03 | DOWN |  |
| AT1G65310 | -2.69 | 8.73E-08 | 1.96E-06 | DOWN | XTH17 |
| ATCG01180 | -2.72 | 1.33E-09 | 4.21E-08 | DOWN |  |
| AT3G30720 | -2.73 | 1.40E-28 | 3.30E-26 | DOWN | QQS |
| AT5G65320 | -2.76 | 5.66E-04 | 4.75E-03 | DOWN | BHLH99 |
| AT2G07629 | -3.03 | 5.60E-05 | 6.43E-04 | DOWN |  |
